# *ISL1* is a major susceptibility gene for classic bladder exstrophy and a regulator of urinary tract development

**DOI:** 10.1038/srep42170

**Published:** 2017-02-08

**Authors:** Rong Zhang, Michael Knapp, Kentaro Suzuki, Daiki Kajioka, Johanna M. Schmidt, Jonas Winkler, Öznur Yilmaz, Michael Pleschka, Jia Cao, Christina Clementson Kockum, Gillian Barker, Gundela Holmdahl, Glenda Beaman, David Keene, Adrian S. Woolf, Raimondo M. Cervellione, Wei Cheng, Simon Wilkins, John P. Gearhart, Fabio Sirchia, Massimo Di Grazia, Anne-Karolin Ebert, Wolfgang Rösch, Jörg Ellinger, Ekkehart Jenetzky, Nadine Zwink, Wout F. Feitz, Carlo Marcelis, Johannes Schumacher, Federico Martinón-Torres, Martin Lloyd Hibberd, Chiea Chuen Khor, Stefanie Heilmann-Heimbach, Sandra Barth, Simeon A. Boyadjiev, Alfredo Brusco, Michael Ludwig, William Newman, Agneta Nordenskjöld, Gen Yamada, Benjamin Odermatt, Heiko Reutter

**Affiliations:** 1 Institute of Human Genetics, University of Bonn, Bonn, Germany; 2 Department of Genomics, Life & Brain Center, University of Bonn, Bonn, Germany; 3 Institute of Medical Biometry, Informatics, and Epidemiology, University of Bonn, Bonn, Germany; 4 Developmental Genetics, Institute of Advanced Medicine, Wakayama Medical University, Japan; 5 Institute of Anatomy, University of Bonn, Bonn, Germany; 6 Department of Women´s and Children´s Health and Center for Molecular Medicine, Karolinska Institutet, Stockholm, Sweden; 7 Department of Pediatric Surgery, University Hospital Lund, Lund, Sweden; 8 Department of Women’s and Children’s Health, Uppsala Academic Children Hospital, Uppsala, Sweden; 9 Department of Pediatric Surgery, Queen Silvias Children’s Hospital, Gothenburg, Sweden; 10 Centre for Genomic Medicine, University of Manchester, Manchester, UK; 11 Paediatric Urology, Royal Manchester Children’s Hospital, Central Manchester University Hospitals NHS Foundation Trust, Manchester, UK; 12 Institute of Human Development, University of Manchester, Manchester Academic Health Science, Centre, Manchester, UK; 13 Royal Manchester Children’s Hospital, Manchester, Manchester, UK; 14 Department of Pediatric Surgery, Capital Institute of Pediatrics, Beijing, People’s Republic of China; 15 Department of Paediatrics and Department of Surgery, Southern Medical School, Faculty of Medicine, Nursing and Health Sciences, Monash University, Clayton, VIC, Australia; 16 Department of Surgery, Beijing United Family Hospital, Beijing, People’s Republic of China; 17 Cabrini Monash University Department of Surgery, Cabrini Hospital, Melbourne, Australia; 18 Department of Epidemiology and Preventive Medicine, School of Public Health and Preventive Medicine, Monash University, Australia; 19 Division of Pediatric Urology, Johns Hopkins School of Medicine, Baltimore, MD, USA; 20 Department of Medical Sciences and Medical Genetics Unit, Città della Salute e della Scienza University Hospital, University of Torino, Torino, Italy; 21 Institute for Maternal and Child Health, IRCCS Burlo Garofalo, Trieste, Italy; 22 Department of Urology and Pediatric Urology, University Hospital of Ulm, Germany; 23 Department of Pediatric Urology, St. Hedwig Hospital Barmherzige Brüder, Regensburg, Germany; 24 Department of Urology, University Hospital Bonn, Bonn, Germany; 25 Division of Clinical Epidemiology and Aging Research, German Cancer Research Center, Heidelberg, Germany; 26 Department of Child and Adolescent Psychiatry and Psychotherapy, Johannes-Gutenberg University, Mainz, Germany; 27 Department of Urology, Pediatric Urology Center, Radboud University Nijmegen Medical Center, Nijmegen, The Netherlands; 28 Department of Genetics, Radboud University Nijmegen Medical Center, Nijmegen, The Netherlands; 29 Translational Pediatrics and Infectious Diseases, Hospital Clínico Universitario de Santiago, Santiago de Compostela, Spain; 30 GENVIP Research Group (www.genvip.org), Instituto de Investigación Sanitaria de Santiago, Galicia, Spain; 31 Genome Institute of Singapore, Singapore; 32 Division of Genomic Medicine, Department of Pediatrics, University of California Davis Medical Center, Sacramento, CA, USA; 33 Department of Clinical Chemistry and Clinical Pharmacology, University of Bonn, Bonn, Germany; 34 Centre for Genomic Medicine, University of Manchester, Manchester, UK; 35 Pediatric Surgery, Astrid Lindgren Children Hospital, Karolinska University Hospital, Stockholm, Sweden; 36 Department of Neonatology & Pediatric Intensive Care, Children’s Hospital; University of Bonn, Bonn, Germany

## Abstract

Previously genome-wide association methods in patients with classic bladder exstrophy (CBE) found association with *ISL1*, a master control gene expressed in pericloacal mesenchyme. This study sought to further explore the genetics in a larger set of patients following-up on the most promising genomic regions previously reported. Genotypes of 12 markers obtained from 268 CBE patients of Australian, British, German Italian, Spanish and Swedish origin and 1,354 ethnically matched controls and from 92 CBE case-parent trios from North America were analysed. Only marker rs6874700 at the *ISL1* locus showed association (*p* = 2.22 × 10^−08^). A meta-analysis of rs6874700 of our previous and present study showed a *p* value of 9.2 × 10^−19^. Developmental biology models were used to clarify the location of ISL1 activity in the forming urinary tract. Genetic lineage analysis of *Isl1*-expressing cells by the lineage tracer mouse model showed *Isl1*-expressing cells in the urinary tract of mouse embryos at E10.5 and distributed in the bladder at E15.5. Expression of *isl1* in zebrafish larvae staged 48 hpf was detected in a small region of the developing pronephros. Our study supports *ISL1* as a major susceptibility gene for CBE and as a regulator of urinary tract development.

The bladder exstrophy-epispadias complex (BEEC; OMIM %600057) represents the severe end of human congenital anomalies of the kidney and urinary tract (CAKUT), and involves the abdominal wall, pelvis, all of the urinary tract, the genitalia, and occasionally the spine and anus. Birth prevalence rates for the most common defect form, classic bladder exstrophy (CBE), range from 1 in 30,000 to 1 in 50,000, varying among North American ethnic groups with the highest prevalence being observed among Native Americans (8 in 100,000) and the lowest among Asians (1 in 100,000)^1^,^2^. Vesicoureteral reflux and obstruction of the ureteropelvic junction are observed frequently in both genders, and cryptorchidism is common in males^3^. Long-term complications are malignancies of the bladder mucosa, with 95% of these malignancies being adenocarcinomas^4^.

To identify susceptibility loci for CBE, we recently performed a genome-wide association study (GWAS) of 110 CBE patients and 1,177 controls of European origin. Here, an association was found with a region of approximately 220 kb on chromosome 5q11.1-q11.2^5^. This region harbors the *ISL1* (ISL LIM homeobox 1) gene. Multiple markers in this region showed evidence for association with CBE, including 84 markers with genome-wide significance. A subsequent meta-analysis using data from a previous GWAS by our group of 98 CBE patients and 526 controls of European origin also implicated the 5q11.1-q11.2 locus in CBE risk^5^,^6^. A total of 138 markers at this locus reached genome-wide significance in the meta-analysis. No other locus in the meta-analysis achieved genome-wide significance.

The present association study followed-up on the most promising genomic regions based on the results of our previous meta-analysis defined by a total of 12 independent markers. One of these markers resides in the *ISL1* region and had previously reached genome-wide significance^5^. The other 11 markers had previously reached *p* values of 10−05 5. The study sample before quality control (QC) steps of the generated genotypes comprised 274 Australian, British, German, Italian, Spanish, and Swedish CBE patients and 1,365 ethnically matched controls, and 110 case-parent trios from North America of European background. To better understand the role of *ISL1* during genito-urinary tract development, we performed expression studies in mouse embryos and zebrafish larvae (zfl).

## Results

### Association study

After performance of QC steps for the generated genotypes we had to exclude six CBE patients and 11 of the ethnically matched controls from the case-control study. Hence the analyzed data set for the case-control study comprised 268 CBE patients of Australian (n = 31), British (n = 40), German (n = 7), Italian (n = 39), Spanish (n = 35), and Swedish (n = 116) origin and 1,354 ethnically matched controls. In controls, there was no evidence for deviation from Hardy-Weinberg equilibrium for any of the 12 SNPs (data not shown). Accordingly we had to exclude 18 of the CBE case-parent trios from North America of European from the transmission disequilibrium test (TDT). Hence the analyzed data set for the TDT comprised 92 case-parent trios from North America of European background. Table 1 shows the results of the association analyses for the 268 cases, the ethnically matched 1,354 controls, the 92 case-parent trios, and the combined meta-analysis. A more detailed description of the results of the TDT is shown in the Supplementary (Supplementary Table 1). A significant result was observed for the marker rs6874700 at chromosome 5q11.2, representing the *ISL1* locus (*p* = 2.22 × 10^−08^). A meta-analysis for the marker rs6874700 of our previous and present study showed a *p* value of 9.2 × 10^−19^. The relative risk (RR) (95% CI) for rs6874700 in this meta-analysis was 1.93 (95% CI, 1.67–2.23) (Table 2).

### Lineage analysis of *Isl1*-expressing cells in mouse embryos

**Figure 1:**
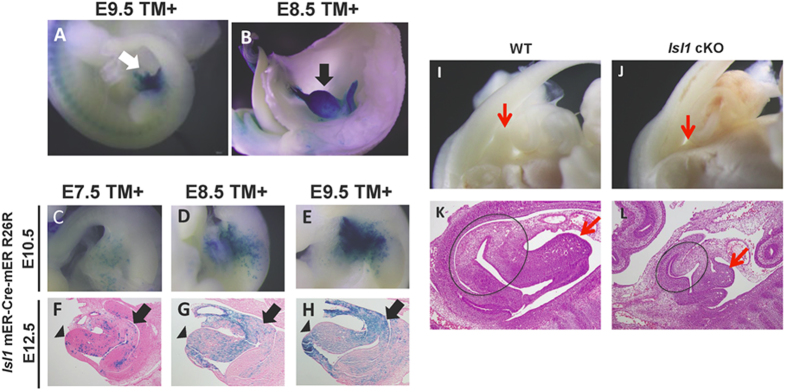
(**A,B**) Lineage analysis of *Isl1*-expressing cells at E10.5 and E15.5 by whole mount lacZ staining; (**A**) Contribution of *Isl1*-expressing cells in mouse embryos at E10.5 (white arrowhead); (**B**) Contribution of *Isl1*-expressing cells in mouse embryos at E15.5 (white arrow). (**C–H)** Lineage analysis of *Isl1*-expressing cells after administration of tamoxifen (TM) at E7.5–E9.5; (**F–H**) Mid-sagittal sections of bladder region at E12.5. The arrows and arrowheads indicate the bladder and genital tubercle, respectively. (**I–L**): Hypoplasia of GT and bladder in Isl1 cKO embryos.

Lineage tracing provides a powerful means of understanding tissue development, homeostasis, and disease, especially when it is combined with experimental manipulation of signals regulating cell fate decisions. In order to address the contribution of Isl1-expressing cells in the developing early genito-urinary tract, we used the Isl1-mER-Cre-mER allele in which sequences encoding a tamoxifen dependent Cre recombinase were inserted into the Isl1 locus^7^. Here Isl1-expressing cells were observed in the early genito-urinary tract of mouse embryo at E10.5 (Fig. 1A, arrow). Isl1-expressing cells later distributed in the bladder at E15.5 (Fig. 1B). To further analyze the involvement of Isl1- expressing cells for the bladder formation, we performed detailed cell lineage analysis by conducting several timing of tamoxifen treatments. An increasing number of Isl1-expressing cells at E7.5 were detected in the early genito-urinary tract and bladder region (Fig. 1C,F). Of note, a large number of Isl1-expressing cells were observed in such regions after the treatment of tamoxifen at E8.5 and E9.5 (Fig. 1D,E,G,H). In addition to the bladder, Isl1-expressing cells also contributed to the genital tubercle region (Fig. 1F–H, arrowheads). To further investigate the contribution of Isl1-expressing cells for caudal body formation, we analyzed the Hoxa3-IRESCre; Isl1Flox/Flox mutant embryos (*Isl1* cKO). Hoxa3 Cre driver mouse line possesses Cre activity in the caudal body regions^8^. *Isl1* cKO showed the hypoplasia of GT (Fig. 1I–L, red arrows) as well as bladder (Fig. 1K,L, circle). The embryonic time dependent Isl1 positive mesenchymal cell population was thus discussed further in Discussion.

### Whole mount *in situ* hybridization (WISH) of zebrafish larvae (zfl)

**Figure 2:**
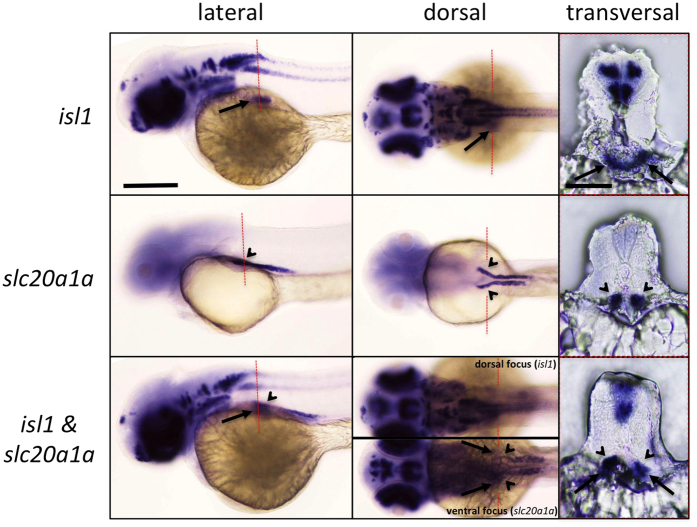
WISH against *isl1* (antisense 3′-untranslated region-probe; upper row), *slc20a1a* (middle row) and both probes combined (lower row) on wild-type zfl at 48 hpf. 48 hpf long-pec stage zfl in lateral (left column) and dorsal view (middle column; same fish; scale bar = 500 μm) in the top row are showing *isl1* expression amongst other previous described regions also at most anterior regions of the proximal convoluted tubular (PCT; arrows). 25 μm transversal slices sections of the same fish are shown in the right column (scale bar = 150 μm). The orientation of sections is shown with red dashed lines in the corresponding whole mount images. The zfl show a clear *isl1* staining in what has previously been described as the developing pancreatic region, and which might well be overlapping with an anterior region of the *slc20a1a* labeled PCT (black arrow heads). The overlap of *isl1* and *slc20a1a* expression at this region is further supported by the fact that no additional stained area could be found there, when we used both *in situ* probes simultaneously for staining. *Isl1* expression could not be detected in the developing cloaca or proctodeum region (not shown).

In contrast to mammals, in early developmental stages of zfl between 48–96 hours post fertilisation (hpf) the pronephros containing two glomeruli plus nephrons, is already fully functional. In adult fish the subsequently developing mesonephros will yet become the final kidneys. In mammals however only the hereafter developing metanephros will become the final kidney^9^. In the early developing zfl, just after gastrulation (tail-bud stage around 10 hpf) first renal progenitors can be detected in bilateral stripes of intermediate mesoderm (IM), located either side of the embryonic midline in between the paraxial mesoderm (PM) and the lateral plate mesoderm (LPM)^10^. These renal progenitor fields give then, from about 10 somite stage, rise to a pair of bilateral nephrons. The pronephros itself is again comprised of a series of discrete cell types, well differentiable approximately from the prim-5 stage on at around 26 hpf^11^,^12^. To test for a potential involvement of *isl1* in the above described renal progenitor development in zfl, our first step was to investigate *isl1* expression at these sides during the respective time points by whole mount RNA *in situ* hybridization (WISH) expression analysis in the early wt zfl between 22 and 60 hpf (corresponding approximately to 26 somite and pec-fin stage). Using an antisense *in situ* probe comprising an UTR region of zfl isl1 cDNA (see methods section^13^,^14^) we could clearly detect *isl1* expression in some region of the developing pronephros for all tested time points mentioned above. At 48 hpf we could clearly detect *isl1* expression at most anterior regions of the proximal convoluted tubular (PCT) regions (Fig. 2), which we co-localized with a previously described *slc20a1a in situ* marker^12^. Long-peg stage zfl at 48 hpf showed *isl1* expression in regions close to the yolk-sac, which have previously been described as the early developing pancreatic region^15^, but do also overlap with *slc20a1a* marker expression here in the most anterior developing nephros region (Fig. 2).

## Discussion

Following-up on the most promising genomic regions based on the results of our previous meta-analysis in a total of 360 CBE patients, we were able to replicate our findings on the *ISL1* harboring region with the marker rs6874700 (*p* = 2.22 × 10^−8^). A meta-analysis for the marker rs6874700 of our previous and present study showed a *p* value of 9.2 × 10^−19^. The relative risk (RR) (95% CI) for rs6874700 in this meta-analysis was 1.93 (95% CI, 1.67–2.23).

Isl1 is a transcription factor, containing LIM domains, which has been described as playing essential roles for the regulation of cell lineage commitment in organogenesis. It is essential for development of heart, kidney, neurons and limb^15^,^16^ however, the role of Isl1 in caudal embryonic region has been left unexplored. The cloaca is the essential embryonic structure to develop the external genitalia and the adjacent mesenchymes (termed as peri-cloacal mesenchyme, PCM)^17^. Cloacal epithelia and the surrounding mesenchyme interaction play a major role in caudal embryonic development. The mesenchyme adjacent to the cloaca receives cloacal derived signals and such mesenchyme includes the anterior part of the PCM (aPCM), which locates in the anterior (upper) part of the cloacal field^17^,^18^.

Our findings in CBE patients for the *ISL1* locus are further supported by lineage analysis of *Isl1*-expressing cells in mouse embryos and mutant mice analysis of Isl1 cKO embryos. Isl1 marks the aPCM, which has been shown to contribute to the embryonic formation of the bladder, rectum, and the external genitalia^19^. As outlined above, many CBE patients present with additional anomalies of the human CAKUT spectrum including vesicoureteral reflux and obstruction of the ureteropelvic junction underlining that CBE affects all of the urinary tract system and not the bladder only. Thus, *Isl1* cell lineages match with the organs reflecting various phenotypes of CBE. In the current study, we found that Isl1 is required for the caudal body formation including bladder and GT. The accurate lineage analyses of Isl1-expressing cells suggest that aPCM cells are specified to the bladder and external genitalia around at E7.5. Hence, the current study revealed the presence of the embryonic-time dependent mesenchymal cell population as sensitive for the current set of genetic mutations. The epithelial-mesenchymal interaction between the epithelia and PCM is necessary to form the major parts of the caudal embryo. During such caudal embryonic development, several signaling pathways such as Bone morphogenetic protein (Bmp) and Sonic Hedgehog (Shh) are activated in the aPCM. On note, Defects of Bmp signals cause the body wall closure defects^20^,^21^. Another growth factor, Shh, is reported as expressed in the early staged cloacal epithelia affecting the adjacent PCM development^17^. It has been also shown that Shh signals relay to modulate mesenchymal Bmp signals^22^. Hence, the current analyses suggest that the signals including Shh, Bmp possibly affecting Isl1 lineage aPCM cells specified at early embryonic stage such as E7.5. Further analyses are required to understanding the molecular mechanisms for the bladder and body wall formation.

Using WISH analysis in early developmental stages of zfl (22–60 hpf), we were able to detect *isl1* expression close to the proximal region of the developing pronephros for all tested time points. Later during development of zfl, the two nephrons fuse with the caudal end of the intestine, attaching at 96 hpf at the cloaca^23^. The pronephros filtrates the blood before delivering the waste to the cloaca for excretion, thus representing very premature structures of an urinary tract^10^. Therefore, even though *isl1* could not be detected in the developing more distal cloaca or proctodeum regions in the zfl we analyzed, proximal expression of *isl1* at regions of these early structures of mammalian urinary tract equivalents in zebrafish provides additional evidence for *ISL1* as a regulator for urinary tract development across species.

We were not able to replicate any of the other 11 regions tested in our single-marker analysis. While we cannot exclude, that the inability to find further evidence for association for these 11 regions is based on a lack in statistical power of our present study, it is more likely, that these regions, respectively markers within these regions, had a higher effect in our previous meta-analysis (‘winner’s curse’) (208 CBE patients in total) than in our extended replication sample (268 CBE patients in total in our case-control study and 92 case-parent trios in our TDT). Alternatively, these regions might have been false positive signals in our previous meta-analysis, which were unrelated to CBE, or only show association with CBE patients of central European origin but not in CBE patient cohorts of different ethnicity. Finally, and most likely, our previous meta-analysis may have failed to identify the possible relevance of other markers and regions, that are actually involved in human CBE formation but were not considered for replication in our follow-up step since they might have obtained *p* values which were not considered worthy of note (e.g. *p* values > 10^-5^)^5^. It is therefore warranted, in order to identify additional markers and regions that might actually be involved in human CBE formation that we perform genome-wide genotyping of the present sample in a separate analysis. It is therefore warranted, in order to identify additional markers and regions that are actually involved in human CBE formation to perform genome-wide genotyping of the present sample in a separate analysis.

Within our replicated region of genome-wide significance and with an achieved *p* value of 1.14 × 10^−10^ in our previous meta-analysis the marker rs2303751 in exon 4 of *ISL1* reaches a score of 2b (scale 1a–6) in “RegulomeDB” (http://regulomedb.org) indicating that rs2303751 resides in a functional region. The marker rs2303751 alters 17 DNA binding motifs according to HaploReg v4.1, including alteration of the position-weight matrix for SMAD4, an essential downstream transcriptional regulator of the TGF beta signaling pathway^24^. In addition, the genomic region encompassing rs2303751 is occupied by EZH2 (enhancer of zeste 2), the major component of the genome regulating Polycomb Repressive Complex 2 (PRC2) and a therapeutic target in human bladder cancer^25^. With EZH2 being involved in human bladder cancer and with SMAD4 playing an essential role for initiating the smooth muscle cell differentiation program during ureter development, further analysis of the rs2303751 comprising region enclosing *ISL1* is warranted^24^,^25^.

In addition, Kitchen *et al*., looking at methylation in low/intermediate grade non-muscle invasive bladder cancer, showed that the *ISL1* genes’ promoter-associated island was more frequently methylated in recurrent and progressive high-grade tumors than their non-recurrent counterparts^26^. Since, Kolarova *et al*., were just recently able to provide certain lines of evidence, that imprinting might play a role in BEEC formation, further studies regarding differences in methylation of the *ISL1* region in CBE patients compared to matched controls are also warranted^27^.

## Conclusion

Our present association study in CBE patients together with functional studies in mouse embryos and zebrafish larvae suggest *ISL1* as a regulator of urinary tract development.

## Materials and Methods

### Ethics statement and subjects

This study was approved by each participating center’s institutional ethics committee. All methods were carried out in accordance with relevant guidelines and regulations. All experimental protocols were approved by the institutional committee of the University of Bonn. Furthermore the study was conducted according to Declaration of Helsinki principles. Written informed consent was obtained from all patients, parents/guardians and the normal controls. Demographic information was collected from both, patients and controls, through a structured questionnaire. Experienced physicians trained in the diagnosis of the BEEC personally recruited all BEEC patients included in this study.

The study sample comprised 274 Australian (n = 36), British (n = 40), German (n = 7), Italian (n = 40), Spanish (n = 35), and Swedish (n = 116) CBE patients and 1,365 ethnically matched controls, and 110 case-parent trios from North America of European background. Peripheral venous blood samples, saliva samples or buccal swab samples were obtained from patients and controls for DNA extraction. More details about the recruitment process for patients and population based respectively phenotype unrelated controls can be found elsewhere and in the Supplementary ‘Materials, Methods’^5^,^6^,^27^.

### Genotyping

For SNP-marker genotyping, we used the Sequenom MALDI-TOF mass-spectrometer (MassArray system), TaqMan^®^ SNP genotyping assays (Life Technologies Europe BV, Netherlands) and conventional Sanger sequencing. Sequenom data were analyzed using the Spectrodesigner Software package (Sequenom, San Diego, CA) and allele peaks were analyzed with the Sequenom Typer Analysis software. TaqMan generated data were analyzed using the ABI 7900HT instrument and the SDS software (v2.4, Applied Biosystems). Sanger sequencing generated data were analyzed using CodonCode Aligner software package (CodonCode Corporation, MA, USA).

### Statistical analysis

Statistical analysis was performed using SAS software (version 9.1). The standard chi-square test was used to test for deviations from Hardy-Weinberg equilibrium. The two-sided Armitage’s trend test was used to compare genotype distributions between cases and controls separately in each case-control subsample. In order to test for association of the risk alleles with CBE in trios from North America, we applied the transmission/disequilibrium test (TDT)^31^. Fixed effects meta-analysis was used to combine samples^32^.

### Embryo collections for lineage analysis of *Isl1*-expressing cells and analysis of knockout mouse embryos

All experimental procedures and protocols were approved by the Committees on Animal Research at Wakayama Medical University and the experiments were carried out in accordance with the approved guidelines. Each mouse strain used in this study Isl1flox/flox conditional mutant allele, Hoxa3-IRESCre, Isl1-mER-Cre-mERm and R26R-LacZ indicator strain was described previously^7^,^8^,^33^. The tamoxifen (TM)-inducible Cre recombinase system removes the floxed sequence from the target genome^31^. TM (Sigma, St Louis, MO, USA) was dissolved in sesame oil at 10 mg/ml and injected into pregrant female by oral administration at a dose of 0.05 mg/g of body weight. The mice for Isl1 positive-lineage analyses used in this study were Isl1-mER-Cre-mER and R26R-LacZ indicator strains. These mice strains were generated as described previously^7^,^34^. TM was administrated from embryonic days (E) 7.5 to E9.5 and the embryos were harvested at E10.5, E12.5 and E15.5, respectively. X-gal staining for the detection of Isl1-expressing cells was performed as previously described^33^,^36^. Briefly, freshly dissected embryos were fixed 1 hour in a solution composed of 1% formaldehyde and 0.2% glutaraldehyde at 4 °C. The embryos were washed with PBS and color development was performed with the developing solution. For histological analysis of the Isl1-expressing cells, the X-gal stained specimens were refixed overnight in 4% paraformaldehyde (PFA)/PBS, dehydrated in methanol and embedded in paraffin. 10 μm serial sections were prepared and stained by Eosin.

For analysis of Hoxa3-IRESCre; Isl1 mutant embryos (hereafter described as Isl1 cKO), we mated Hoxa3-IRESCre; Isl1Flox/+ with Isl1Flox/Flox, and subsequently harvested at E13.5. Embryonic specimens were fixed overnight in 4% paraformaldehyde (PFA)/PBS, dehydrated in methanol, and embedded in paraffin. Serial sections (6-μm thick) were prepared for hematoxylin/eosin (H&E) staining.

### Whole mount *in situ* hybridization (WISH) of zebrafish larvae (zfl)

All experimental procedures and protocols were carried out in accordance with the approved national guidelines. Zfl for the presented experiments were maintained and staged as described elsewhere^14^,^37^. Mixed Tübingen long fin (TL) and Ekkwill (EK) strain wild-type (wt) embryos were used for all experiments. All embryos were gained by natural fish spawning set up in the mornings and raised at 28 °C in Danieau (30%) medium, following local and international animal procedure standards. To analyse *isl1*-expression in zfl mRNA WISH was performed in embryos of different ages (22 hpf, 48 hpf and 60 hpf). We mainly followed a WISH standard protocol using an anti-Dig Ab-dilution of 1:10:000^37^. The RNA *in-situ* probe was designed for binding at the 5′ untranslated (UTR) region of zf *isl1* using the following forward primer (CATCATctcgagAGAGTGACATCGACCAGCCTGCTTTCC) and the reverse primer (CATCATggatccGAAATTCCCACACAGCTTGTGGC) on cDNA of 1 dpf whole zfl. The resulting amplified PCR product of the probe was then cloned into SK(-)pBluescript^®^ using BamHI and XhoI sites and sequenced. We used the DIG RNA Labeling Kit (SP6/T7) (Roche; product number: 11175025910) to transcribe an antisense (T3) and a sense probe (T7) as negative control (not shown) from the above plasmid. Final imaging of the WISH stained larvae fish was performed using an Eclipse upright microscope (Nikon) equipped with a 10x and 40x objectives (NA = 0.25 and 0.65 respectively) and a DS-Vi1 digital colour camera run by NIS-Element software (Nikon).

## Additional Information

**How to cite this article:** Zhang, R. *et al*. *ISL1* is a major susceptibility gene for classic bladder exstrophy and a regulator of urinary tract development. *Sci. Rep.* **7**, 42170; doi: 10.1038/srep42170 (2017).

**Publisher's note:** Springer Nature remains neutral with regard to jurisdictional claims in published maps and institutional affiliations.

## Supplementary Material

Supplementary Information

## Figures and Tables

**Table 1:** Results of the association analyses for cases and controls, the case-parent trios, and the combined meta-analysis.

SNP	Chromosome/Region	Position	Risk/other allele	Meta-analysis Cases (n = 268) vs. controls (n = 1,354) (AUS/GB/DE/IT/ES/SE) RR (95% CI)	TDT Case-parent trios (n = 92) (North America) RR (95% CI)	Meta-analysis Cases (n = 268), Controls (n = 1,354), Case-parent trios (n = 92) RR (95% CI) *p* values	
rs1475601	1q31.3	194,721,655	G/A	1.11 (0.60–2.05)	0.88 (0.32–2.41)	1.04 (0.61–1.76)	0.884
rs72748303	1q32.2	208,973,633	A/G	1.54 (0.95–2.52)	0.71 (0.23–2.25)	1.37 (0.87–2.15)	0.170
rs79145914	4p14	39,490,706	G/A	1.26 (0.74–2.14)	1.75 (0.51–5.98)	1.33 (0.82–2.15)	0.254
rs7689350	4q13.3	76,207,570	A/C	0.98 (0.70–1.38)	2.13 (0.92–4.92)	1.10 (0.80–1.51)	0.568
**rs6874700**	**5q11.2**	**50,701,750**	**A/T**	**1.80 (1.44–2.25)**	**1.61 (1.07–2.41)**	**1.75 (1.44–2.13)**	**2.2 × 10^−08^**
rs1514351	6q12	68,694,074	C/T	1.20 (0.96–1.50)	0.91 (0.60–1.39)	1.13 (0.92–1.37)	0.239
rs117622209	6q22.1	118,108,159	C/T	1.12 (0.64–1.96)	1.20 (0.37–3.93)	1.13 (0.69–1.88)	0.623
rs56189125	7p14.3	28,855,348	G/A	1.05 (0.09–11.78)	1.67 (0.61–4.59)	1.56 (0.61–3.96)	0.353
rs57086087	8p11.21	40,301,811	T/C	1.07 (0.80–1.44)	1.42 (0.84–2.39)	1.15 (0.89–1.48)	0.301
rs10119066	9p24.1	7,509,895	T/G	0.99 (0.66–1.50)	1.60 (0.52–4.89)	1.05 (0.71–1.55)	0.806
rs16917077	9p21.1	31,532,212	C/T	1.39 (0.74–2.59)	1.38 (0.55–3.42)	1.38 (0.83–2.32)	0.215
rs1514921	12q21.2	79,481,756	C/T	1.21 (0.89–1.64)	1.35 (0.72–2.53)	1.23 (0.94–1.62)	0.136

**Table 2:** Results of our previous meta-analysis and the presently tested sample for the marker rs6874700.

SNP	Chromosome/Region	Position	Risk/other allele	Previous meta-analysis Draaken *et al*.^5^ RR (95% CI)	Replication RR (95% CI)	Present meta-analysis RR (95% CI) *p* values	
rs6874700	5q11.2	50,701,750	A/T	2.17 (1.75–2.70)	1.75 (1.44–2.13)	1.93 (1.67–2.23)	9.2 × 10^−19^
