# Supplementary material for: ISL1 is a major susceptibility gene for classic bladder exstrophy and a regulator of urinary tract development
Source: Sci Rep. 2017 Feb 8;7:42170. doi: 10.1038/srep42170 (PMC5296905; doi:10.1038/srep42170)
Supplement: Supplementary Information [file srep42170-s1.docx]

**Supplementary Information to Zhang et al.**

**Title:** *ISL1* is a major susceptibility gene for classic bladder exstrophy and a regulator of urinary tract development

Rong Zhang^1,2,*^, Michael Knapp^3,*^, Kentaro Suzuki^4^, Daiki Kajioka^4^, Johanna M. Schmidt^1,5^, Jonas Winkler^5^, Öznur Yilmaz^5^, Michael Pleschka^5^, Jia Cao^6^, Christina Clementson Kockum^7^, Gillian Barker^8^, Gundela Holmdahl^9^, Glenda Beaman^10^, David Keene^11^, Adrian S. Woolf^12,13^, Raimondo M. Cervellione^11^, Wei Cheng^14,15,16^, Simon Wilkins^17,18^, John P. Gearhart^19^, Fabio Sirchia^20^, Massimo Di Grazia^21^, Anne-Karolin Ebert^22^, Wolfgang Rösch^23^, Jörg Ellinger^24^, Ekkehart Jenetzky^25,26^, Nadine Zwink^25^, Wout F. Feitz^27^, Carlo Marcelis^28^, Johannes Schumacher^1^, Federico Martinón-Torres^29,30^, Martin Lloyd Hibberd^31^, Chiea Chuen Khor^32^, Stefanie Heilmann-Heimbach^1,2^, Sandra Barth^1,2^, Simeon A. Boyadjiev^32^, Alfredo Brusco^20^, Michael Ludwig^33^, William Newman^34^, Agneta Nordenskjöld^6,35^, Gen Yamada^4^, Benjamin Odermatt^5^, Heiko Reutter^1,36^, *authors contributed equally

^1^ Institute of Human Genetics, University of Bonn, Bonn, Germany; ^2^ Department of Genomics, Life & Brain Center, University of Bonn, Bonn, Germany; ^3^ Institute of Medical Biometry, Informatics, and Epidemiology, University of Bonn, Bonn, Germany; ^4^ Developmental Genetics, Institute of Advanced Medicine, Wakayama Medical University, Japan; ^5^ Institute of Anatomy, University of Bonn, Bonn, Germany; ^6^ Department of Women´s and Children´s Health and Center for Molecular Medicine, Karolinska Institutet, Stockholm, Sweden; ^7^ Department of Pediatric Surgery, University Hospital Lund, Lund, Sweden; ^8^ Department of Women's and Children's Health, Uppsala Academic Children Hospital, Uppsala, Sweden; ^9^ Department of Pediatric Surgery, Queen Silvias Children’s Hospital, Gothenburg, Sweden; ^10^ Centre for Genomic Medicine, University of Manchester, Manchester, UK; ^11^ Paediatric Urology, Royal Manchester Children’s Hospital, Central Manchester University Hospitals NHS Foundation Trust, Manchester, UK; ^12^ Institute of Human Development, University of Manchester, Manchester Academic Health Science, Centre, Manchester, UK; ^13^ Royal Manchester Children’s Hospital, Manchester, Manchester, UK; ^14^ Department of Pediatric Surgery, Capital Institute of Pediatrics, Beijing, People's Republic of China; ^15^ Department of Paediatrics and Department of Surgery, Southern Medical School, Faculty of Medicine, Nursing and Health Sciences, Monash University, Clayton, VIC, Australia; ^16^ Department of Surgery, Beijing United Family Hospital, Beijing, People's Republic of China; ^17^ Cabrini Monash University Department of Surgery, Cabrini Hospital, Melbourne, Australia; ^18^ Department of Epidemiology and Preventive Medicine, School of Public Health and Preventive Medicine, Monash University, Australia; ^19^ Division of Pediatric Urology, Johns Hopkins School of Medicine, Baltimore, MD, USA; ^20^ Department of Medical Sciences and Medical Genetics Unit, Città della Salute e della Scienza University Hospital, University of Torino, Torino, Italy; ^21^ Institute for Maternal and Child Health, IRCCS Burlo Garofalo, Trieste, Italy; ^22^ Department of Urology and Pediatric Urology, University Hospital of Ulm, Germany; ^23^ Department of Pediatric Urology, St. Hedwig Hospital Barmherzige Brüder, Regensburg, Germany; ^24^ Department of Urology, University Hospital Bonn, Bonn, Germany; ^25^ Division of Clinical Epidemiology and Aging Research, German Cancer Research Center, Heidelberg, Germany; ^26^ Department of Child and Adolescent Psychiatry and Psychotherapy, Johannes-Gutenberg University, Mainz, Germany; ^27^ Department of Urology, Pediatric Urology Center, Radboud University Nijmegen Medical Center, Nijmegen, The Netherlands; ^28^ Department of Genetics, Radboud University Nijmegen Medical Center, Nijmegen, The Netherlands; ^29^ Translational Pediatrics and Infectious Diseases, Hospital Clínico Universitario de Santiago, Santiago de Compostela, Spain; ^30^ GENVIP Research Group (www.genvip.org), Instituto de Investigación Sanitaria de Santiago, Galicia, Spain; ^31^ Genome Institute of Singapore, Singapore; ^32^ Division of Genomic Medicine, Department of Pediatrics, University of California Davis Medical Center, Sacramento, CA, USA; ^33^ Department of Clinical Chemistry and Clinical Pharmacology, University of Bonn, Bonn, Germany; ^34^ Centre for Genomic Medicine, University of Manchester, Manchester, UK; ^35^ Pediatric Surgery, Astrid Lindgren Children Hospital, Karolinska University Hospital, Stockholm, Sweden; ^36^ Department of Neonatology & Pediatric Intensive Care, Children's Hospital; University of Bonn, Bonn, Germany

**Supplementary Materials and Methods**

**Sample description**

This study was approved by each participating center’s institutional ethics committee. All methods were carried out in accordance with relevant guidelines and regulations. All experimental protocols were approved by the institutional committee of the University of Bonn. Furthermore the study was conducted according to Declaration of Helsinki principles. Written informed consent was obtained from all patients, parents/guardians and the normal controls. Demographic information was collected from both patients and controls through a structured questionnaire. Experienced physicians trained in the diagnosis of the BEEC personally recruited all BEEC patients included in this study. The study sample comprised 274 Australian (n=36), British (n=40), German (n=7), Italian (n=40), Spanish (n=35), and Swedish (n=116) CBE patients and 1,365 ethnically matched controls, and 110 case-parent trios from North America of European background. Peripheral venous blood samples, saliva samples or buccal swab samples were obtained from patients and controls for DNA extraction.

Australian sample (n=36). In total 36 Australian CBE patients of European background were enrolled at the Royal Children’s Hospital, Melbourne, Australia. Since no ethnically matched Australian controls were available for case-control comparison we used 152 phenotype unrelated inhouse controls of European origin. Therefore, besides the assessment of demographic information on all Australian CBE patients, a multidimensional scaling (MDS) analysis using genome-wide genotyping data generated by Infinium OmniExpressExome-8 BeadChips (San Diego, California, USA) was performed and compared with genome-wide genotypes of the 152 phenotype unrelated inhouse controls of European origin generated by the same Infinium OmniExpressExome-8 BeadChips (data not shown). Based on this analysis 5 CBE patients of Australian origin were excluded from further case-control comparison, since they were outliers in the MDS analysis. For case-control comparison we used the same 152 phenotype unrelated inhouse controls of European origin sampled at the Institute of Human Genetics in Bonn.

British sample (n=40). In total 40 British CBE patients were enrolled at the Department of Paediatric Urology, Royal Manchester Children’s Hospital, Central Manchester University Hospitals. For case-control comparison we used 80 ethnically matched healthy British controls sampled at the Centre for Genomic Medicine at University of Manchester.

The German cases were pooled with the Australian cases and compared to the above mentioned 152 phenotype unrelated inhouse controls of European origin (German sample n=7). In total 7 German CBE patients were enrolled at the University Hospitals of Bonn, Ulm and Regensburg.

Italian sample (n=40). In total 40 Italian CBE patients were enrolled through the Department of Medical Sciences and Medical Genetics Unit, Città della Salute e della Scienza University Hospital, at the University of Torino. For case-control comparison we used 85 ethnically matched phenotype unrelated Italian controls sampled also through the Department of Medical Sciences and Medical Genetics Unit at the University Hospital of Torino.

Spanish sample (n=35). In total 35 Spanish CBE patients were enrolled through the author HR while visiting the Spanish self-help organization in 2004 and 2006 (www.extrofia.info/asafex). For case-control comparison we used 871 Spanish controls recruited through the ESIGEM study consortium ([www.esigem.org](http://www.esigem.org)) as part of the European research consortium on meningococcal disease (meningococcal Genetics Consortium). This study was approved by the `Clinical Research Ethics Committee of Galicia` (Decision No 2005/295).

Swedish sample (n=116). In total 116 Swedish CBE patients were recruited from the Pediatric Surgery Departments in Stockholm, Gothenburg, Uppsala and Lund, Sweden. DNA samples from 177 placentas, acquired after normal delivery of healthy newborns in 2006 at the Karolinska University Hospital, where used as controls.

North American sample (n=110 case-parent trios). In total 110 CBE patients and their healthy parents from North American with European background were sampled at the Pediatric Urology Clinic at the James Buchanan Brady Urological Institute, Johns Hopkins University School of Medicine.

**Supplementary Table 1**

| **SNP** | **Chromosome/**  **Region** | **Position** | **Risk/**  **other allele** | **T** | **NT** | **TDT** | **TDT**  ***p* values** |
| --- | --- | --- | --- | --- | --- | --- | --- |
| rs1475601 | 1q31.3 | 194,721,655 | G/A | 8 | 7 | 0.07 | 0.796 |
| rs72748303 | 1q32.2 | 208,973,633 | A/G | 7 | 5 | 0.33 | 0.564 |
| rs79145914 | 4p14 | 39,490,706 | G/A | 7 | 4 | 0.82 | 0.366 |
| rs7689350 | 4q13.3 | 76,207,570 | A/C | 17 | 8 | 3.24 | 0.072 |
| **rs6874700** | **5q11.2** | **50,701,750** | **A/T** | **61** | **38** | **5.34** | **0.021** |
| rs1514351 | 6q12 | 68,694,074 | C/T | 46 | 42 | 0.18 | 0.670 |
| rs117622209 | 6q22.1 | 118,108,159 | C/T | 6 | 5 | 0.09 | 0.763 |
| rs56189125 | 7p14.3 | 28,855,348 | G/A | 10 | 6 | 1 | 0.317 |
| rs57086087 | 8p11.21 | 40,301,811 | T/C | 34 | 24 | 1.72 | 0.189 |
| rs10119066 | 9p24.1 | 7,509,895 | T/G | 8 | 5 | 0.69 | 0.405 |
| rs16917077 | 9p21.1 | 31,532,212 | C/T | 11 | 8 | 0.47 | 0.491 |
| rs1514921 | 12q21.2 | 79,481,756 | C/T | 23 | 17 | 0.9 | 0.343 |
